# Barriers to adherence to antiretroviral treatment in a regional hospital in Vredenburg, Western Cape, South Africa

**DOI:** 10.4102/sajhivmed.v17i1.476

**Published:** 2016-09-30

**Authors:** Ivo N. Azia, Ferdinand C. Mukumbang, Brian van Wyk

**Affiliations:** 1 School of Public Health, University of the Western Cape, South Africa

## Abstract

**Background:**

South Africa currently runs the largest public antiretroviral treatment (ART) programme in the world, with over 80% of people living with HIV and/or AIDS on ART. However, in order to appreciate the benefits of using ART, patients are subject to uncompromising and long-term commitments of taking at least 95% of their treatment as prescribed. Evidence shows that this level of adherence is seldom achieved because of a multilevel and sometimes interwoven myriad of factors.

**Objective:**

We described the challenges faced by patients on ART in Vredenburg with regard to ART adherence.

**Methods:**

A descriptive qualitative research design was used. Eighteen non-adhering patients on ART in the Vredenburg regional hospital were purposefully selected. Using a semi-structured interview guide, we conducted in-depth interviews with the study participants in their mother tongue (Afrikaans). The interviews were audio-taped, transcribed verbatim and translated into English. The data were analysed manually using the thematic content analysis method.

**Results:**

Stigma, disclosure, unemployment, lack of transport, insufficient feeding, disability grants and alternative forms of therapy were identified as major barriers to adherence, whereas inadequate follow-ups and lack of patient confidentiality came under major criticisms from the patients.

**Conclusion:**

Interventions to address poverty, stigma, discrimination and disclosure should be integrated with group-based ART adherence models in Vredenburg while further quantitative investigations should be carried out to quantify the extent to which these factors impede adherence in the community.

## Introduction

### Background

South Africa has one of the largest HIV epidemics globally. Of the estimated 35 million people living with HIV and/or AIDS (PLWHA) in the world by the end of 2014, South Africa accounted for over 6.8 million cases, with more than 370 000 new infections and 240 000 deaths linked to AIDS.^1^ In 2007, the South African government made a commitment towards combating its AIDS epidemic by implementing a National Strategic Plan, which had as one of its principal objectives, the provision of antiretroviral (ARV) medications to 80% of all people eligible for treatment by 2011.^2^ South Africa now runs the largest public antiretroviral treatment (ART) programme in the world, with over 80% of PLWHA eligible for ART already initiated on treatment in the country.^3,4^

Although ART does not cure HIV/AIDS, it is known to significantly reduce the viral loads of PLWHA and thus forms an integral part of the comprehensive approaches used in curbing AIDS epidemics worldwide.^5^ However, to appreciate the benefits associated with the use of ARV drugs, patients are required to focus on long-term commitments of taking at least 95% of the treatment as prescribed, given that poor adherence is linked with multidrug resistance and even death resulting from opportunistic infections.^6,7,8^

Practically, adherence to ART is a complex and dynamic process. Several barriers to adherence to ART have already been established by numerous studies in both developed and developing countries.^9,10,11^ Demographic variables such as age, gender and ethnicity are known to be inconsistent in predicting good adherence to ART.^12^ Literature has consistently revealed that socio-economic variables such as unemployment, poverty, food insecurity and transport costs are implicated in poor adherence to ARV medications.^13,14^ Socially related variables, such as stigma and discrimination, disclosure and lack of social support, have also been identified to negatively influence the adherence of patients to ART.^15,16,17^ Human resource constraints in developing countries are reported to cause congestion and long waiting times in ART centres and thus deter many ART users from accessing treatment.^18^ Poor healthcare provider practices, including inadequate counselling sessions, abuse of patient confidentiality, lack of adherence follow-ups and drug stock-outs have been demonstrated as major health systems barriers to adherence.^19,20^ The complex nature of ART regimens with a high burden of pills, food and fluid restrictions is also a well-established barrier to adherence.^21^ Patient-related factors such as forgetfulness, side effects, feeling better, mistrust and myths regarding ARV medication are well documented for inhibiting many patients from adhering to treatment.^22,23,24^ The use of substances such as alcohol and illicit drugs while on ART has been found to promote non-adherence to ART in many patients.^25^ In sub-Saharan Africa, the increasing use of traditional concoctions, holy water, as well as prayers as a cure for HIV and/or AIDS is reported as barrier to ART adherence.^26,27^

### Research problem

Poor ART adherence rates (taking less than 95% of medications as prescribed) are empirically proven to have negative outcomes such as medication failure, viral mutations and the development of drug resistance, which further complicate the already limited second- and third-line treatment options available for patients.^6,7,8^ To better understand and address issues of inadequate ART adherence, factors that influence adherence have also been investigated.^16,23,28^ However, there is a paucity of behavioural research on adherence to ART in the peri-urban/semi-rural settings of the Western Cape Province. This information could be relevant in planning and implementing tailored programmes and strategies to address problems faced by patients receiving ART in Vredenburg and similar settings.

## Research aim and objectives

### Aim

The aim of the study was to describe the barriers to adherence to ART among patients in the Vredenburg regional hospital in the Western Cape.

### Specific objectives

The specific objectives of the study were:

- to describe the experiences of HIV and/or AIDS patients in Vredenburg about accessing ART
- to describe the social, structural, economic and cultural factors that influence adherence to ART in Vredenburg.

#### Definitions of key concepts

**Antiretroviral therapy:** Standard ARV therapy consists of a combination of ARV drugs to maximally suppress the HIV virus and stop the progression of HIV to AIDS and also prevent the onward transmission of HIV in the population.^29^

**Adherence**: Is defined as the ability of patients to follow treatment plans, take medications at prescribed times and frequencies and also follow restrictions regarding lifestyles, food and other medications.^29^

**Non-adherence**: Is defined as the inability of patients to follow treatment plans, take medications at prescribed times and frequencies and also follow restrictions regarding lifestyles, food and other medications.^29^

## Methodology

### Study design

A descriptive qualitative study approach was used. According to Pope and Mays,^30^ the qualitative approach is well suited for studies exploring health behaviours. The choice of a qualitative approach was motivated by the fact that the researchers were interested in obtaining a deeper understanding of issues surrounding patients’ adherence or non-adherence to ART in Vredenburg, based on their experiences and perspectives.

### Study population and sampling

All PLWHA who were registered in the ART programme in the Vredenburg general hospital for more than 6 months prior to the inception of this study constituted the study population. The non-adhering patients were identified through patients’ medical records by health practitioners working in the ART programme. After identifying the non-adhering patients, those who could be contacted and were also physically and mentally able to undergo an in-depth interview were approached by a doctor or a nurse working in the ART programme for their possible recruitment into the study. We held several briefing sessions with the doctor and the non-adhering patients about the nature of the study and how they were going to participate and contribute in the study. Twenty-two patients initially accepted to take part in the study, but finally only 18 were interviewed. The demographic characteristics of the participants are summarised in Table 1.

**TABLE 1:** Demographic characteristics of the participants.

Demographic variable	Representations	*n*
Age representation (in years)	Average	41
	Range	18–63
	Mode	38
Age distribution (in years)	10–19	1
	20–29	2
	30–39	7
	40–49	3
	50–59	3
	60–69	2
Gender	Male	5
	Female	13
Level of education	No formal education	4
	Primary school	4
	Attempted high school	9
	Completed high school	1
Marital status	Single	13
	Married	2
	Divorced	1
	Widow or widower	2
Parental status	Participants who had children	10
	Participants who had no children	8
Employment status	Employed	4
	Not employed	14
Racial distribution	Black people	6
	Mixed-race people	12
	White people	-
Duration of time on antiretroviral treatment	Between 6 months and 1 year	12
	Greater than 1 year but less than 2 years	4
	Greater than 2 years	2

### Data collection

We used a semi-structured interview guide to conduct the in-depth interviews. The interview guide was used throughout the study to ensure standardisation and consistency across the interviews. The interviews were conducted in the language of the participants’ choice in a suitable private setting where the respondents could talk freely. The interviewers were guided by the participants’ responses to probe for the emerging themes. Each interview session lasted between 55 and 65 minutes.

#### Data analysis

The data were analysed manually using the thematic content analysis guided by the five stages of thematic analysis as illustrated by Pope et al.^31^ namely: familiarisation stage, theme identification stage, indexing stage, charting stage, and the mapping and interpretation stages. Using these five steps of thematic content analysis, we discovered themes and subthemes, winnowed the themes to a manageable few and constructed hierarchies of themes.^32^ A consensus was reached among the investigators on the themes identified after an interactive discursive process. Finally, the subthemes obtained were deductively represented under economic, social, health team and system, therapy-related, patient-related and cultural factors.

#### Trustworthiness

The trustworthiness of the study was addressed through enforcing credibility, transferability, dependability and conformability as guided by Lincoln and Guba.^33^ The investigators held several meetings during which the approach to data collection and interpretation was carefully selected. Dependability in the results was ensured by sharing the transcripts with experienced researchers at the University of the Western Cape who independently reviewed the coding frame with the data transcripts. We later on reconciled the differences in coding decisions.

#### Ethical considerations

Ethical approval was sought from the Senate Research Committee of the University of the Western Cape. Participation in the study was voluntary, and the participants were free to withdraw from the study without being penalised in any way. A participant information sheet with a brief explanation of the purpose and nature of the study was provided to the participants and explained in their language of choice. They were asked to sign a consent form before participating in the study. Anonymity in the study was ensured by keeping all data secure throughout the study.

## Results

The data obtained from the interviews with the 18 participants were organised around the following themes: (1) Economic barriers, (1) Social barriers, (3) Health team and system barriers, (4) Therapy-related barriers, (5) Patient-related barriers and (6) Cultural barriers. Table 2 shows the themes, subcodes and codes that emerged from the data analysis.

**TABLE 2:** Themes, subcodes and codes obtained from the data analysis.

Theme	Subtheme	Codes
Economic barriers	Poverty	Lack of money for personal needs and transport
		Food shortage
		Disability grant
	Unemployment	Lack of a source of income or loss of employment
Social barriers	Unintended disclosure of HIV status	Fear of unintended disclosure when seen at antiretroviral treatment centres
	Stigma and discrimination	Humiliation
		Rejection
	Social support	Emotional distress and lack of family support
		Lack of moral and spiritual support
Health system barriers	Treatment literacy	Misunderstanding of prescriptions
		Poor counselling sessions
	Quality of care	Long waiting times and lack of follow-up
		Lack of doctors
		Abuse of patient confidentiality
Therapy-related barriers	Side effects	Loss of appetite, bodily discomfort and rashes
		Drowsiness, depression and fatigue
	Feeling well	Continued risky behaviours
Patient-related barriers	Substance abuse	Alcohol and substance abuse
	Emotional distress	Forgetfulness and confusion
	Fatigue	Hard labour and tiredness
Cultural barriers	Use of traditional medicine while on antiretroviral treatment	Simultaneous use of traditional medicines and antiretroviral treatments to cure HIV and/or AIDS
	Religious beliefs	The belief that traditional medicine could cleanse HIV internally
		Believe in holy water and prayers as a cure for HIV/AIDS

### Economic barriers

The economic factors that were identified by respondents to interfere with their ability to take ART as prescribed were poverty and unemployment, lack of money for transport, lack of food and disability grants.

#### Poverty and unemployment

Many respondents reported that they sometimes failed to take their ART because they are poor and unemployed; therefore, they could not raise enough money to buy food and eat properly before taking the medication. This was captured in the excerpt below:

‘I don’t work, I’m not eating properly, I don’t have food, I don’t have money and those things and the baby is … you see for me why would I drink the pills if it doesn’t help me at the end of the day.’ [Female respondent, aged 38 years]

#### Lack of money for transport

It was reported that taxi fare to and from the ART centre at that time of this study was R36. A 63-year-old female respondent complained that the Vredenburg clinic was too far for her to pick up her medication; so she sometimes missed her ART appointments because she did not always have money for transport. Another female respondent expressed her challenges of securing transport in the following words:

‘There are sometimes that I will need to go to the clinic and have no money so I will ask for assistance from my children and sometimes when I have no money for transport I hike to the ART clinic.’ [Female respondent, aged 60 years]

#### Disability grants

It was reported that some patients deliberately discontinued taking their ARTs to enable them lower their CD4 counts and become very sick. The patients then used their low CD4 count and the severity of their sickness to persuade the doctors to qualify them for a disability grant. This is what a 33-year-old female participant reported in connection with securing a disability grant:

‘Maybe you will qualify for a Grant when the doctor will see you don’t have an income and you can’t work and you are weak. So most of them, they just uh…Stop with the medication because they want to receive a Grant.’ [Female respondent, aged 33 years]

### Social barriers

Socially related factors that were identified by the respondents to hamper adherence to ART were fear of unintended disclosure when using the ART clinic, fear of unintended disclosure when seen interacting with community HIV-care providers, fear of unintended disclosure leading to the secret trade on ART, fear of being stigmatised and discriminated against by community members and finally, the fear of being humiliated by some community members if a patient’s HIV status was unintentionally disclosed.

#### Fear of unintended disclosure of HIV status

A number of patients reported that they discontinued accessing the ART clinic because they were afraid that their status could be unintentionally disclosed if they were seen queuing up at the ART clinic regularly. One 60-year-old female respondent pointed out that if someone was often seen in the community having lengthy discussions with any HIV-care provider, that person’s HIV status was automatically assumed and this caused some patients to miss important appointments with HIV-care providers:

‘So if they see someone from the community speaking with these ART workers for a long time, they will assume that the person speaking with them is HIV positive.’ [Female respondent, aged 60 years]

Another 33-year-old female respondent mentioned in the quotes captured below that some patients in the community stopped going to the ART clinic because they did not want others to know about their HIV status. As a result of the fear of disclosure, the patients were going behind the backs of the healthcare providers and instigating those patients accessing the ART clinic to sell to them some of their medications collected from the ART clinic:

‘I heard that other people saying maybe if, I am working now and I have a friend who is HIV now and who does not want to come to the doctor then maybe I should steal the tablets here and then I take them to her to buy.’ [Female respondent, aged 33 years]

#### Stigma and discrimination

Stigma and discrimination were identified from the data as social barriers to medication adherence. Some patients admitted discontinuing their treatment when they started drinking to cope with the emotional depression that they had developed from being stigmatised and discriminated against by their friends and some family members. The following experiences were given by a 25-year-old female respondent on how she was stigmatised and discriminated against by her friends and some of her family members:

‘Some of my friends…, they ignore me or if I came to her she will say, oh, I want to go there or there or say something like that. So the experience was that some of the people do not want you because of the sickness that you have. They do not want to drink in your cups or eat out of your bowl. My mother was doing that, because I was having my own glass, my own cup and my own plate, it made me feel like I never wanted to live anymore. So that is why I started drinking.’ [Female respondent, aged 25 years]

‘I stopped taking my pills for about 2 weeks because my boyfriend humiliated me openly that I was an HIV patient.’ [Female respondent, aged 32 years]

### Health system barriers

Health system–related factors that were found to obstruct medication adherence were the poor treatment literacy, the lack of follow-up of patients, dissatisfaction about the quality of the ART services provided, lack of confidentiality in the way patients’ HIV results were handled, long waiting times at ART clinics and the fear of picking up other infections from other patients in the outpatient department.

#### Poor treatment literacy

A 36-year-old male respondent confessed that he did not take his pills accurately because he was often confused and unable to recall exactly the time and the right number of pills to be taken. This is what he said:

‘Then I asked her sister hmm, why do you give me this one in the morning and this one in the afternoon, you say when I am going to sleep? She said ‘I learned that from the doctor that you must drink the two in the morning and when you go to sleep say before 12 pm.’ [Male respondent, aged 36 years]

The 38-year-old male respondent also reported how he sometimes skipped taking his pills at night before sleeping because of exhaustion or because he overslept:

‘Wednesday, Thursday I was working so hard, sometimes when I come back home and sit down, I fall asleep immediately.’ [Male respondent, aged 38 years]

#### Lack of follow-up

A respondent testified of missing his ART schedules because of inadequate follow-up by the nurse in charge at the rehabilitation unit. Consequently, his medication supply ran out without the nurse realising and he missed his pills for 4 days before they were finally replenished. This is what he said in connection to the lack of follow-up on his ARVs by the nurse in charge:

‘When I was leaving, all the pills were finished. So I left, I was there for 4 days, without them.’ [Male respondent, aged 38 years]

### Therapy-related barriers

Two therapy-related factors that hindered adherence to ART identified by the respondents were side effects and feeling better after commencing treatment.

#### Side effects

A 38-year-old female respondent was the only respondent who stopped taking her ART for 3 weeks because she got fed up with the pruritus (itches) she experienced when she started taking the treatment. The respondent said she experienced side effects and discomfort in the following ways:

‘I said doctor, I just stopped my pills for three weeks I got fed up of with these pills, you know what I am saying and now I experienced the itching and all those things and I was stressed out because of all those things.’ [Female respondent, aged 38 years]

#### Feeling better

Feeling better was identified as a barrier to medication adherence in some patients. A 25-year-old female respondent testified that when she experienced improvements in her health after commencing treatment, she stopped taking her medications and carried on socialising with her friends as she did prior to her initiation in the ART programme. This is what she had to say:

‘I was not taking them, and the weekend I was not taking them, because I was for a year and two months without, without my pills…. It was on my birthday when I started drinking with my friends, July month.’ [Female respondent, aged 25 years]

### Patient-related barriers

The respondents pointed out the following patient-related factors to have negatively affected their adherence to treatment: alcohol abuse and smoking, emotional distress, forgetfulness, confusion, fatigue and hard labour.

#### Alcohol abuse and smoking

Alcohol is known to impair peoples’ reasoning and can therefore negatively affect adherence. Most respondents who started drinking and smoking while on ART reported that heavy drinking and smoking hindered them from taking their ART as prescribed. While those who drank and smoked only occasionally during weekends and birthday parties also reported that they missed taking their ART after drinking heavily. A 25-year-old female respondent made the following testimony:

‘I was on my own, started drinking and smoking again and that is why I dropped my pills and now I am back on treatment.’ [Female respondent, aged 25 years]

#### Emotional distress

Emotional distress can disrupt peoples’ moods and their ability to concentrate on taking their ART. According to some respondents, they did not concentrate on taking their ART as prescribed because they were emotionally distressed either by a broken relationship or by stigmatisation and rejection at home. A 38-year-old male respondent had this to say with regards to being emotionally distressed while on ART:

‘Something that happened between us; so we were not married… So I decided to leave her alone. I could not use that medication and I came to the doctor and told him but-but this woman, what she has done to me I cannot take it any longer so I, I started drinking heavily.’ [Male respondent, aged 38 years]

#### Forgetfulness

Forgetfulness was found by some respondents to have caused them to sometimes miss doses sometimes. A 38-year-old male respondent, in the quote below, explained how he missed taking his ART because he sometimes forgot about them especially when he overslept and did not take note of the reminder from his alarm clock or cell phone:

‘You see that happened to me, not so far away from 7 o’clock, it happened once with me, in the morning at 7 o’clock I drank my pills but later at the same time it was 7 o’ clock again…I forgot and it was to 8.’ [Male respondent, aged 38 years]

### Cultural barriers

#### The simultaneous use of concoctions from traditional healers while on antiretroviral treatment

A culturally related factor that was found to have been negatively affecting adherence to ART was the simultaneous use of concoctions from traditional healers while on ART. It was reported that some respondents were still using concoctions prepared by traditional healers for cleansing their bodies internally as well as taking ART medications. A 38-year-old female respondent made this testimony on her simultaneous use of ART and concoctions from traditional healers:

‘No, no… I am using the mixtures, it is the mixtures that must make me like this, and it is cleaning me. That is my concern.’ [Female respondent, aged 38 years]

A 38-year-old female respondent mentioned that she was of the opinion that one could take mixtures from traditional healers while on ART as long as one informed both parties about using both types of treatments. This is how she expressed her opinion regarding the use of traditional healers’ concoctions while on ARTs:

‘You have to take both of them, and you have to tell the doctor and your traditional healers that you are also on ARVs.’ [Female respondent, aged 38 years]

## Discussion

### Financial constraints

In our study, many respondents missed hospital appointments and/or their doses either because they were unable to raise enough money for transport fares or because they were unable to buy food and eat properly to enable them to take their medications as prescribed. This is consistent with literature that patients on higher incomes have less difficulty with adherence compared to patients with low-income levels.^34^ Although ART programmes are presently making them more affordable to users by providing them free of charge at the points of delivery, in South Africa, unemployment, transportation cost, childcare, school fees and lack of food as well as loss of earnings are still major concerns that hinder patients from adhering optimally to treatment.^35^

### Disclosure of HIV status

Disclosure of one’s HIV status and the readiness to live with the disease is a difficult process. A number of patients in our study skipped their doses or missed hospital appointments because they were afraid that their HIV status could unintentionally be disclosed if they were seen in queues at the ART clinics regularly. These findings are common to those obtained in a South Indian study in which patients who did not disclose their HIV-positive status, travelled long distances to obtain anonymous treatment and hid or skipped pills to ensure that family members and employers did not know about their HIV status.^36^ Nonetheless, disclosure was also found to play both a negative and positive role in adherence in this study, given that those patients who intentionally disclosed to family members, friends or religious groups, gained some form of social, physiological, economical and spiritual support, which motivated them to step up their adherence to ART as compared to those who did not.

### Stigma and discrimination

Stigma was found to be pivotal in driving discrimination against people living with AIDS in the community and thus negatively influenced adherence in our study. Stigma and discrimination have been reported as two important variables well documented for greatly contributing to low rates of disclosure and thus poor adherence to ART.^37^ In this study, stigma was found to have provoked discrimination, rejection and the isolation of people living with AIDS. HIV stigma created the emotional distress that pushed some patients to skip doses and to take up heavy drinking as a way of providing some self-comfort.

### Inadequate follow-up and dissatisfaction in antiretroviral treatment services

A study from Tanzania found that long waiting hours (>10 hours) in a hospital setting to access ART, reduced clinic attendance and consequently hindered adherence.^38^ However, in our study long waiting times was not a major concern but inadequate follow-ups and lack of confidence in health officials were reported as major concerns with a potential of interrupting patients’ abilities to access ART treatment in future.

### Side effects and discomfort

While therapy-related variables such as the complex nature of ARV regimens, with a high number of pills, foods and fluids restrictions and the difficulty of fitting regimens into daily living activities was reported in Malangu^28^ to have negatively influenced adherence in Pretoria South. In our study only one respondent reported that she stopped taking her medications for 2 weeks because of the unbearable itches that she experienced. This suggests that side effects, pill burden, and food and fluid restrictions are not major barriers to adherence in Vredenburg.

### Feeling better and the hope to live longer

Feeling better in this study was like a double-edged sword that positively and negatively influenced adherence in some patients. While some ART users believed in the treatment because they were sure to regain their health after the treatment, others took advantage of the improvement in their health and deliberately stopped taking their treatment in order to carry on with their past risky social lifestyles. The hope to live longer and the disastrous consequences of poor adherence to ART, experienced by some patients, was found to have motivated them to step up their adherence. This result matches those obtained by Watt et al.^39^ in a study in Tanzania in which men were motivated to adhere to treatment because it provided them with an opportunity to return to their work, support their families and care for their children.

### Substance abuse, forgetfulness and fatigue

In this study, we also found that most of our respondents who started drinking heavily and smoking while on ART reported that heavy drinking and smoking hindered them from taking their ART as prescribed. Substance abuse in patients taking ART has been noted to have far-reaching consequences on their ability to adhere to therapy, as it makes it difficult for them to receive any form of social and family support, which can motivate them to adhere properly to therapy.^35^ Although alcohol and smoking were found to negatively influence adherence, other factors associated with substance abuse such as forgetfulness, fatigue and confusion were also recorded to have negatively influenced adherence in this study.

### The use of alternative therapy

The use of traditional medicines while on ART has been previously reported as a potential barrier to ART adherence in Uganda.^26^ In this study, many respondents also reported to have been using concoctions prepared by traditional healers for cleansing their bodies. Others said that they could also take mixtures from traditional healers while on ART, if they informed both parties about the different treatments that they were using. This suggests that the concomitant use of traditional mixtures while on ART could be a barrier to ART adherence in the community. It is worth mentioning that some respondents were still visiting traditional healers and religious prophets for prayers and other solutions to their HIV problem. These findings are in line with those from an Ugandan study,^26^ which found that believing in divine healing was found to be a barrier to ART adherence in 1.2% of the patients in the study.

### Limitations of the study

Time and financial constraints hindered the researchers from conducting all the interviews. Thus, the interviews were also conducted by research assistants. This could also have influenced the quality of the data collected and also the results of the study. Secondly, the study did not include some of the adhering patients in the same facility of Vredenburg who may have had similar experiences as those included in the study. In that case, the investigators would have explored what made them adherent despite the challenges or how they overcame the challenges. Therefore, we might have missed valuable lessons for incorporation into adherence messages to help those who were non-adherent.

### Recommendations

This study revealed that patients in Vredenburg are still facing significant barriers to ART adherence, such as stigma, discrimination, poverty and disclosure. Therefore, poverty, stigma and disclosure are recommended to remain at the forefront of the ART programme implementation in Vredenburg, while further quantitative investigations are also recommended to quantify the extent to which the above-mentioned factors impede ART adherence in Vredenburg.

## Conclusion

Although barriers to ART adherence, such as financial constraints, substance abuse, alternative therapy use, disclosure and recklessness in healthcare services obstructed adherence in this study, HIV and/or AIDS has been a stigmatised illness since its onset in the early 1980s, and these results highlight that poverty, disclosure and stigma are yet to dissipate in the community of Vredenburg.
